# Th17细胞在肿瘤中的研究进展

**DOI:** 10.3779/j.issn.1009-3419.2011.11.10

**Published:** 2011-11-20

**Affiliations:** 100038 北京，首都医科大学附属北京世纪坛医院肿瘤研究室 Cancer Research Laboratory, Capital Medical University, Beijing Shijitan Hospital, Beijing 100038, China

**Keywords:** Th17淋巴细胞, 肿瘤免疫, 肿瘤微环境, Th17 lymphocyte, Tumor immunology, Tumor microenvironment

## Abstract

Th17细胞是近年来被鉴定出的一类新型CD4阳性T细胞亚群。与传统的Th1、Th2细胞不同，它以分泌IL-17为主要特征，并参与自身免疫性疾病和慢性炎症的发病过程。最近的证据表明Th17细胞也参与肿瘤免疫，然而这些细胞究竟是促进肿瘤的发展或是抑制肿瘤的发展尚不清楚。本综述总结了有关Th17细胞在肿瘤中作用的最新资料，分析Th17细胞在肿瘤中的特性和作用，以利于未来设计更有效的肿瘤治疗方案。

## Abstract (en)

The Th17 cells, identified recently as a novel CD4^+^ T cell lineage, are characteristic of their production of IL-17 and distinct from Th1 and Th2 lineages. Their involvement in autoimmune and chronic inflammation diseases has been well observed. Recent evidence suggests that Th17 cells are also involved in tumor immunology. However, it remains unclear that how these cells regulate immune responses to tumor growth. In this review, we summarize the most recent findings about the biologics of the Th17 cells in tumor development with a hope of providing new insights into future development of effective new cancer immunotherapies.

CD4^+^T细胞在免疫系统中发挥着重要作用，它们能辅助B细胞产生抗体，增强和维持CD8^+^T细胞的免疫应答，调节巨噬细胞的功能及免疫反应的幅度和持久性等。如果其数目或功能发生改变，就有可能使机体易患各种疾病^[1]^。

传统CD4^+^T细胞分为Th1和Th2两类，前者表达特征转录因子T-bet，分泌IFN-γ，主要促进细胞内感染的病原体的清除; 后者表达特征转录因子GATA-3，分泌IL-4、IL-5和IL-13，主要促进寄生虫（如蠕虫）感染的清除。最近又有两类不同于Th1和Th2的细胞加入其行列，其中一类为Treg细胞（regulatory T cells或调节性T细胞），表达特征转录因子Foxp3，分泌TGF-β，主要维护机体的免疫平衡; 另一类为Th17细胞，表达转录因子ROR-γt，除特征性地分泌IL-17外，还分泌IL-10、IL-21、IL-22等多种因子，主要在自身免疫病、感染性疾病以及移植排斥反应中发挥重要作用^[2]^。Th17细胞在肿瘤免疫中的作用近年来已倍受关注，本文就Th17细胞与肿瘤研究相关的生物学特性中的最新进展作一综述。

## 1 Th17细胞在肿瘤中的数量变化

肿瘤动物模型和多种肿瘤患者的外周血、瘤组织、骨髓、脾等能检测到高水平的Th17细胞。例如，与正常鼠相比，B16黑色瘤鼠的血液、骨髓、脾及瘤组织中的Th17细胞数量明显增多，在头颈部肿瘤、MCA纤维肉瘤及前列腺癌鼠模型中，也观察到高水平的Th17细胞^[3]^。在人的恶性肿瘤中，高水平Th17细胞可出现在晚期卵巢癌、胰腺癌及肾癌组织及外周血中^[3]^。

然而，在某些类型的肿瘤患者中，其外周血Th17细胞数量并不增多，但在其它体液、肿瘤组织中却明显增多。例如，Th17细胞在肺癌患者的恶性胸腔积液中含量明显高于外周血，且数量增多预示患者的生存期延长^[4]^。早期肝癌患者外周血中的Th17细胞比例明显降低，但其瘤组织中的Th17细胞数量却明显增加^[5]^。Sfanos等^[6]^在对前列腺癌浸润CD4^+^T细胞亚群表型分析中，发现存在Th17细胞的漂移，并与前列腺癌的Gleason分级呈负相关。

有些研究^[7-9]^也指出，Th17细胞在早期的肿瘤组织中比例明显升高，但随着疾病的进展，肿瘤浸润的Th17细胞和IL-17水平反而降低，不过发生这一变化的原因目前尚不清楚。

除上述所提到的肿瘤类型外，高水平的Th7细胞也出现在头颈部肿瘤^[10]^、口腔鳞状细胞癌^[11]^、膀胱癌^[12]^、结肠癌^[13]^及中枢神经系统肿瘤如胶质瘤^[14, 15]^和小脑髓母细胞瘤^[16]^等恶性肿瘤的瘤组织，表明Th17细胞浸润是恶性肿瘤的一个普遍特性。

## 2 肿瘤患者外周血Th17细胞的临床意义

有研究^[17]^表明外周血中Th17细胞的数量变化与肿瘤的恶性程度有关，并且其变化可能用于肿瘤的临床分期并预示肿瘤的治疗效果。局限期小细胞肺癌患者外周血中含有较多的能产生IL-17、IFN-γ及IL-4的CD62Llow CD4^+^T效应性细胞（Teff），Teff大部分是Th17细胞，且Teff的数量与患者生存期呈正相关，即长期存活的患者保持很高的Teff/Treg比值，复发性患者常显示Teff/Treg比值降低，说明Th17细胞起免疫保护作用，而Treg细胞则起免疫抑制作用，故作者认为一种治疗小细胞肺癌有希望的策略是增加Th17细胞和降低Treg细胞。与人表皮生长因子受体2（human epidermal growth factor receptor-2, HER-2）阴性乳腺癌患者相比，Th17细胞在HER-2阳性乳腺癌患者外周血中的数量明显下降，经曲妥珠单抗治疗后，其Th17细胞比例增加。而Treg细胞在HER-2阳性乳腺癌患者与HER-2阴性乳腺癌患者外周血中的比例却无统计学差异。Horlock等^[18]^认为检测血中Treg和Th17细胞在患者对某种治疗是否有反应方面能提供有益的指示作用。

此外，胃癌患者外周血中Th17细胞的比例增高，与胃癌的临床分期密切相关^[19]^。Th17细胞在宫颈上皮内瘤和子宫颈癌患者的血液中占有很高的比例，且增高的Th17细胞与临床分期、淋巴结转移及血管浸润密切相关^[20]^。在有激素抗性非骨转移的前列腺癌患者中，主动全细胞免疫治疗前外周血中的Th17细胞与疾病进展期呈负相关，提示Th17细胞可预测免疫治疗的效果^[21]^。

## 3 肿瘤浸润Th17细胞的生物学特性

### 3.1 肿瘤浸润Th17细胞的特征

肿瘤浸润的Th17细胞是一类记忆型细胞，但不同的研究报告在有关Th17细胞分子标志物谱的表达上存在一定的差异。Su等^[13]^发现肿瘤浸润的Th17细胞，既表达记忆型标志物CCR7^+^、CD62L^dim/-^、CD45RO^+^，也表达CCR2、CCR4、CCR5、CCR6、CCR7、CXCR3等趋化型受体，但不表达细胞毒相关的标记分子，如CD56、颗粒酶A、Fas配体及抑制分子PD-1。另外，它们还表达属于CD4^+^CD25^+^Treg细胞标志的CTLA-4、CD25。但Kryczek等^[9]^报道，肿瘤浸润的Th17细胞不表达CCR2、CCR5、CCR7，而表达高水平的CXCR4、CCR6、CD61和CD49等分子标志物。但无论如何，表达的这些分子标志物都可能参与Th17细胞的迁移，并与其滞留在肿瘤微环境中有关。Th17细胞另一特征是产生CCL20，而该特征可促进其在肿瘤微环境中的扩增^[22]^。

肿瘤浸润的Th17细胞极少表达HLA-DR、CD25及颗粒酶B，提示Th17细胞是一类非传统的效应性T细胞，即其并非是通过颗粒酶B途径发挥效应作用^[9]^。Th17细胞极少表达抑制免疫的分子如PD-1、B7-H1受体和Foxp3，提示Th17细胞是不同于Treg细胞和PD-1^+^T细胞的一类细胞^[9]^。

肿瘤微环境中的IL-17产生T细胞主要是Th17细胞，少量的IL-17（< 1%）可由CD8^+^T细胞产生^[9]^。肿瘤浸润Th17细胞除分泌大量IL-17外，还分泌其它细胞因子，如IFN-γ、TNF-α、IL-8、IL-10、TGF-β等^[13, 23]^。在几乎所有的肿瘤中都发现有既分泌IL-17又分泌IFN-γ的Th17细胞（Th17/Th1）的存在。不过，它们在肿瘤微环境中究竟起何种作用还需要进一步研究。可见，不同肿瘤类型其Th17细胞的表面标志和分泌特性有所差异，这种差异是否与特定的肿瘤微环境有关，尚有待证实。

### 3.2 肿瘤浸润Th17细胞的功能

Th17细胞和初始CD4^+^T细胞的共培养证实，其可促进初始CD4^+^T细胞的增殖，说明Th17细胞具有免疫调控的作用^[13]^。另外，Th17细胞与Treg细胞有重叠的表型特征，表明Th17细胞有可塑性^[13]^。在与肿瘤细胞相互作用方面，Th17细胞或IL-17对卵巢癌细胞的增殖和凋亡没有直接作用，提示Th17细胞对肿瘤的作用是间接的^[9]^。实验表明，肿瘤微环境中的Th17细胞与Th1型趋化因子CXCL9和CXCL10呈正相关，与Th2型趋化因子CXCL12和CCL22呈负相关，与IFN-γ产生的T细胞（包括IFN-γ^+^CD4^+^T细胞、IFN-γ^+^CD8^+^T细胞、IFN-γ^+^IL-17^+^T细胞）及NK细胞呈正相关，与Treg细胞呈负相关，提示Th17细胞与NK细胞介导的天然免疫及T细胞介导的适应性免疫相关^[9]^。

Th17细胞向肿瘤组织的迁移是依赖CCR6/CCL20途径的^[10]^。Th17细胞分泌的IL-8能募集中性粒细胞，并促进其释放GM-CSF、TNF-α和IFN-γ来发挥效应。反过来，活化的中性粒细胞又能通过CCL20/CCR6依赖的方式来募集Th17细胞^[24]^。Th17细胞也能增强肿瘤组织表达CCL20和CCL2，后者能促进树突状细胞及活化的CD4^+^和CD8^+^T细胞在肿瘤组织的聚集，增强机体的抗肿瘤免疫应答^[25]^。

### 3.3 Th17细胞的抗肿瘤和促肿瘤作用

Th17细胞可通过IL-17和IFN-γ来刺激CXCL9和CXCL10的产生，后者能募集Th1细胞及NK细胞到肿瘤微环境中，从而促进机体的抗肿瘤保护性免疫^[9]^。静脉注射免疫原性弱的B16-F10黑色素瘤的IL-17A缺陷小鼠比野生型鼠更易发生肺黑色素瘤，用肿瘤特异性Th17细胞治疗成瘤鼠则能阻止肿瘤的发展，用抗体阻断IFN-γ后，Th17细胞的抗肿瘤免疫并未受影响，这种Th17细胞仍保持其原有的细胞因子分泌特性而没有转变为Th1细胞表型，并显示出比Th1细胞更强的抗肿瘤免疫效能。进一步的研究^[25]^表明，这是由于Th17细胞能诱发出极强的由CD8^+^T细胞参与的抗肿瘤免疫应答。Alvarez等^[26]^组建了一种疫苗FC-CD40L，即用腺病毒载体把编码CD40L的基因导入由树突状细胞和肿瘤细胞融合的细胞中。这种疫苗能在罹患B细胞淋巴瘤的鼠体内引出极强的Th17细胞免疫应答，使肿瘤消退，患瘤鼠的生存期延长。另外，Th17细胞还能通过非IL-17依赖方式发挥免疫调节作用，包括通过某种依赖IFN-γ的机制来发挥抗肿瘤作用^[22]^。

然而，也有证据^[27]^表明，Th17细胞有促进肿瘤生长和转移的作用。Th17细胞的产物IL-17与骨髓瘤细胞高表达的IL-17受体结合可促进骨髓瘤细胞的增殖并增强骨髓瘤细胞与骨髓基质的粘附，用IL-17R抗体则可拮抗IL-17的促骨髓瘤细胞增殖作用。Th17细胞的产物IL-17和IL-22能抑制Th1细胞介导的抗肿瘤免疫应答^[27]^。也有实验^[5]^证实Th17细胞的水平与肿瘤组织中的微血管密度呈正相关，提示Th17细胞可能通过促进血管生成而导致肿瘤的发展。

Th17与肿瘤发生发展的关系，可能取决于不同Th17细胞的来源或所处的状态。有资料^[15]^显示，把来自正常小鼠脾脏的Th17细胞（nTh17）或罹患胶质瘤鼠脾脏的Th17细胞（gTh17）和胶质瘤细胞系GL261同时经颅内或皮下注入免疫适能的小鼠后，发现输注nTh17细胞的荷瘤鼠生存期明显延长，瘤体内有高水平的IFN-γ和TNF-α及低水平的IL-10和TGF-β; 而输注gTh17细胞的荷瘤鼠生存期明显缩短，瘤体内有高水平IL-10和TGF-β及低水平的IFN-γ和TNF-α。

由此可见，Th17细胞在不同阶段、不同类型的肿瘤中，由于受肿瘤微环境的影响而可能处于不同的状态，从而使其发挥促进或抑制肿瘤生长的作用。

## 4 肿瘤微环境对Th17细胞的作用

肿瘤微环境（包括肿瘤细胞和间质细胞及其分泌的各种细胞因子）有调节Th17细胞分化或增殖的作用，但不同肿瘤类型，其趋化及募集Th17细胞的方式和性质有所不同。

### 4.1 间质细胞或肿瘤细胞对Th17细胞的诱导分化和调节作用

肿瘤微环境中的间质细胞包括巨噬细胞和树突状细胞，它们均可对Th17细胞起诱导分化和调节作用。如骨髓瘤患者骨髓中的树突状细胞，可通过直接接触的方式来调节Th17细胞的扩增^[28]^。体外实验表明，活化的单核细胞和肝癌相关的巨噬细胞均可诱导循环中的记忆性T细胞扩增为Th17细胞。尽管前者的作用更强，但两者所产生的Th17细胞表型特征极其相似。同时，如果抑制活化的单核/巨噬细胞介导的炎性反应就可明显地降低肿瘤浸润的Th17细胞数量及肿瘤的生长^[29]^。虽然肿瘤微环境中的巨噬细胞或树突状细胞能诱导记忆型T细胞分化成Th17细胞，但却不能诱导初始T细胞分化成Th17细胞^[9]^。

另外，肿瘤细胞本身也可直接调节Th17细胞。例如，在无B淋巴瘤细胞的情况下，用IL-1β/IL-6或脂多糖处理CD4^+^T细胞，则Th17细胞的产生增强; 当CD4^+^T细胞与B淋巴瘤细胞共培养时，这种Th17细胞产生增强的效果就会减弱; 如果用抗CD70抗体或抗CD80/86抗体阻断CD27-CD70或CD28-CD80/86的相互作用则可恢复脂多糖诱导Th17细胞产生的效果，表明B淋巴瘤细胞有特异性抑制Th17细胞产生的作用^[30]^。

### 4.2 细胞因子对Th17细胞的调节作用

肿瘤微环境中的各类细胞所分泌的多种细胞因子可募集、调节和促进Th17细胞的分化和增殖^[8, 11]^。肿瘤细胞及肿瘤相关成纤维细胞通过分泌RANTEs和MCP-1来募集外周血中的Th17细胞到肿瘤微环境中; 通过分泌一些炎性介质如IL-6、IL-1β、IL-23、TGF-β等来促进Th17细胞的扩增^[13]^。血管免疫母细胞T细胞淋巴瘤患者瘤组织中含有大量的肥大细胞，这些细胞能分泌IL-6，从而形成有利于Th17细胞分化和增殖的微环境^[31]^。肺癌患者恶性胸腔积液中的促炎因子如IL-1β、IL-6、TGF-β都能促进初始CD4^+^T细胞向Th17细胞分化，其中趋化因子CCL20和CCL22能募集外周血中的Th17细胞^[4]^。Sfanos等^[6]^在对不同前列腺癌患者的个体研究中发现，存在Th17细胞高表达的患者，其Treg则低表达，并指出决定这一现象的关键因素是IL-6。进展期小细胞肺癌患者外周血中含较多Treg细胞，而局限期小细胞肺癌患者外周血中含有较多的Th17细胞，这种偏向Th17细胞而非Treg细胞的分化与肿瘤微环境中IL-23高表达密切相关^[17]^。

### 4.3 Treg细胞对Th17细胞的作用

Th17细胞与Treg细胞表面的大部分趋化受体均相同，Th17细胞与Treg细胞在许多肿瘤组织中均同时存在，二者的动态平衡可能与机体发生适当强度的免疫应答密切相关。Crome等^[32]^报道初始和记忆Treg细胞既能抑制Th17细胞的增殖又能抑制IL-6、IL-8、IL-17和IL-22的产生。Treg细胞可通过Stat3依赖的机制来抑制Th17细胞介导的免疫应答反应，Stat3缺失的Treg细胞不能抑制Th17细胞介导的炎性反应，但仍能抑制Th1和Th2介导的炎性反应^[33]^。也有报道^[9]^认为肿瘤浸润的Treg细胞可通过其它机制（如adenosinergic pathway）来抑制Th17细胞的产生。

另外，Treg细胞可调节Th17细胞分泌细胞因子的状况。在胶质瘤发展的早期，Th17细胞有抑制肿瘤的作用，这种Th17细胞主要分泌IL-17、IFN-γ及少量的IL-10;在肿瘤发展的晚期，Treg细胞的数量大大增加，其结果使Th17细胞转化为能促进肿瘤发展的细胞，这种Th17细胞除仍主要分泌IL-17外，还分泌大量的IL-10及少量的IFN-γ^[15]^。

## 5 Th17细胞的可塑性

CD4^+^T细胞在不同细胞因子环境中可分化为Th1、Th2、Treg和Th17四种细胞亚群，在一定条件下，各Th细胞亚群之间可以互相转化，从而使机体的各种免疫应答处于精细的平衡状态。越来越多的证据表明，已分化的Th17细胞具有可塑性，在适当的刺激下，会转化为其它亚群。体内分离的Th17细胞能在IFN-γ和IL-12的诱导下转变成Th1/Th17细胞，且能稳定地表达特征转录因子RORγt和T-bet^[34]^。Th17细胞在正常鼠内能维持其表型，但在淋巴细胞减少的鼠体内则不能维持其表型而转变为Th1细胞^[35]^。这也说明新生的Th17细胞需要微环境的帮助来维持其表型。

体外培养的肿瘤浸润Th17细胞在OKT3和同种外周血单核细胞的刺激下可分化为能产生IFN-γ的Th1细胞、Treg细胞及双阳性细胞Th17/Th1和Th17/Treg; 反复的OKT3刺激能使源自Th17细胞的Treg拥有潜在的抑制功能，且这种Treg细胞即使在有利于Th17细胞分化的条件下，如IL-1β、IL-6和IL-23存在的条件下，也不能恢复成Th17细胞，表明这种转化的Treg细胞具有一定的自稳性^[36]^。Hoechst等^[37]^报道从人外周血分离的CD14^+^HLA-DR^-/low^髓源性抑制细胞（myeloid-derived suppressor cell, MDSC）不仅能诱导初始CD4^+^T细胞向Treg细胞方向分化，而且还能促进已分化的Th17细胞转化为Treg细胞，中间经历一个IL-17^+^Foxp3^+^双阳性细胞阶段，其中TGF-β在这一转变中起着重要作用。但目前尚未有体内证据表明Th17细胞可转化为Treg细胞。

总之，Th17细胞是近年来新发现的一类新型CD4^+^T细胞亚群，与Th1、Th2和Treg细胞亚群不同，有其独立的分化发育途径。尽管我们对Th17细胞的分化发育、调控机制及功能有了更深刻的理解，但其在肿瘤中的作用目前仍了解有限，所观察到的结果多存在相互矛盾之处。Th17细胞及其相关的细胞因子究竟是起促进肿瘤还是抑制肿瘤的作用，这在更大程度上取决于其所处的微环境; Th17细胞的作用还随着致病因素、肿瘤的类型和疾病进展阶段的不同而有所差异; 再者Th17细胞既能通过依赖IL-17途径又能通过非依赖IL-17途径来发挥肿瘤免疫调节作用。研究已证实Th17细胞具有可塑性，但其生理意义目前尚不清楚，哪类因子维持和重塑Th17细胞尚需进一步确定。可见，Th17细胞在肿瘤中的作用非常复杂，对其在肿瘤发生发展中的作用尚需进行深入的探讨。不过，我们相信随着对Th17细胞研究的深入和其机制的阐明，新的有助于肿瘤诊断和治疗的研究成果也将随之产生。
